# Analysis of the Differential Gene and Protein Expression Profiles of Corneal Epithelial Cells Stimulated with Alternating Current Electric Fields

**DOI:** 10.3390/genes12020299

**Published:** 2021-02-20

**Authors:** Bhavani S. Kowtharapu, Jyoti Damaraju, Nitesh Kumar Singh, Josefin Ziebart, Rainer Bader, Dirk Koczan, Oliver Stachs

**Affiliations:** 1 L V Prasad Eye Institute, Kallam Anji Reddy Campus, Banjara Hills, Hyderabad 500034, India; jyotidamaraju@outlook.com; 2 Department of Ophthalmology, Rostock University Medical Center, 18057 Rostock, Germany; oliver.stachs@uni-rostock.de; 3 Centre for Cellular and Molecular Biology, Hyderabad 500039, India; nitesh@ccmb.res.in; 4 Biomechanics and Implant Technology Research Laboratory, Department of Orthopedics, Rostock University Medical Center, 18057 Rostock, Germany; josefin.ziebart@med.uni-rostock.de (J.Z.); rainer.bader@med.uni-rostock.de (R.B.); 5 Institute for Immunology, Rostock University Medical Center, 18057 Rostock, Germany; dirk.koczan@med.uni-rostock.de

**Keywords:** corneal epithelial cells, electric fields, alternating current, microarray, antibody array

## Abstract

In cells, intrinsic endogenous direct current (DC) electric fields (EFs) serve as morphogenetic cues and are necessary for several important cellular responses including activation of multiple signaling pathways, cell migration, tissue regeneration and wound healing. Endogenous DC EFs, generated spontaneously following injury in physiological conditions, directly correlate with wound healing rate, and different cell types respond to these EFs via directional orientation and migration. Application of external DC EFs results in electrode polarity and is known to activate intracellular signaling events in specific direction. In contrast, alternating current (AC) EFs are known to induce continuous bidirectional flow of charged particles without electrode polarity and also minimize electrode corrosion. In this context, the present study is designed to study effects of AC EFs on corneal epithelial cell gene and protein expression profiles in vitro. We performed gene and antibody arrays, analyzed the data to study specific influence of AC EFs, and report that AC EFs has no deleterious effect on epithelial cell function. Gene Ontology results, following gene and protein array data analysis, showed that AC EFs influence similar biological processes that are predominantly responsive to organic substance, chemical, or external stimuli. Both arrays activate cytokine–cytokine receptor interaction, MAPK and IL-17 signaling pathways. Further, in comparison to the gene array data, the protein array data show enrichment of diverse activated signaling pathways through several interconnecting networks.

## 1. Introduction

Endogenous electric fields (EFs) and wound currents are generated spontaneously in many tissues following injury and are present at the wound site during the process of wound healing [1]. Dynamic changes in wound-generated electric currents demonstrate that electrical signaling is an active response to injury [2]. Many types of cells respond to weak direct current (DC) EFs with a galvanotropic response (polarized shifting of cellular orientation in an EF), which is followed by a galvanotaxic response (migration of a cell in an EF) [3]. Different cell types from the same tissue can respond differently to the same electrical signal. Peculiar differences in Ca^2+^, Na^+^, and K^+^ channels, surface charges, and specific membrane resistance may contribute to the inherent directional movement of cells during applied EFs [4]. In the cornea, injury-induced corneal wound currents directly correlate with the wound healing rate. In physiological conditions, the human cornea shows small outward currents (0.07 μA/cm²), which simultaneously increase up to 5-fold upon wounding (0.41 μA/cm²) for approximately 6 h [5]. The human corneal epithelium contains an active Na^+^/K^+^-ATPase transport system, with an inward flow of sodium ions and active outward transport of Cl^−^ ions across the stroma and epithelium, establishing a trans-epithelial potential difference. Wounding disrupts this potential difference and consequently leads to the flow of Na^+^ and K^+^ into the wound from the surrounding tissue, generating a laterally orientated physiological EF that can direct orientation and migration of corneal epithelial cells and enhance the epithelial wound healing rate by controlling the division axis in epithelial cells [6,7].

The behavior of corneal epithelial cells and keratocytes varies in applied DC EFs, where epithelial cells, at field strength of 400 V/m, migrate in the cathodal direction and stromal fibroblasts (600 V/m) migrate in the anodal direction [8]. Applied external EF stimulations also influence the axis of corneal epithelial cell division, and in the presence of small EFs (e.g., 100 V/m), cultured corneal epithelial cell migration, orientation display serum, growth factor and substrate dependence in vitro [9,10]. Application of high EFs, for example 250 V/m, is necessary to observe cathodal migration of corneal epithelial cells in serum-free culture conditions. Further, human and bovine corneal epithelial cells show similar migration patterns in the applied small EFs [11,12]. The strength of the EFs in the vicinity of the lesion also influences the rate of re-epithelialization [13], while increased wound field strengths enhance corneal wound epithelialization. Studies on the role of wound-induced electrical currents show that Cl^−^ and Na^+^ are the major components of electric currents in rat corneal wounds. In the resting, non-wounded cornea, Na^+^ is the main component of ionic transport, whereas Cl^−^ is an important component of the endogenous EF at the wound edges. These findings allowed researchers to test clinically approved pharmacological agents such as aminophylline, ascorbic acid, and furosemide to control the Cl^−^ flow, thereby modulating electric currents in wounds [14]. The same approach in the form of chloride-free or aminophylline eye drops has been shown to enhance healing of damaged human corneas by offering bioelectric stimulation without electrodes [5]. At the cellular level, corneal injury causes significant changes in distribution and expression pattern of the calcium-activated chloride channel CLC2 in the human corneal epithelium. The time course of CLC2 mRNA upregulation correlates with increased Cl^−^ flux, which sequentially reaches its maximum within 1 h after wounding [2]. Furthermore, applied EFs also influence the regeneration of nerves after injury [15] and wound-generated endogenous EFs also control the rate of nerve sprouting, nerve growth, and regeneration, along with epithelial wound healing [16,17].

All of the literature reports so far regarding the application of external EFs in corneal wound healing have reported on the application of DC EFs. However, in certain cell types, the application of DC EFs has been shown to negatively affect cell viability and proliferation when compared to alternating current (AC) EFs [18,19], suggesting cell survival benefits of AC EFs [20]. Furthermore, it is known that the exposure of cells to AC EFs significantly enhances intracellular calcium signaling and also alters various cellular functions [7,21,22]. DC is the continuous unidirectional flow of charged particles, which results in electrode polarity. However, on the other hand, AC is the continuous bidirectional flow of charged particles without electrode polarity, resulting in the repetitive stimulation of the target cells [23] and also minimizing electrode corrosion [24]. Further, electric stimulation is known to enhance regeneration-associated gene (RAG) expression and regulate cell behavior by activating many intracellular signaling pathways and shows great promise in disease treatment and wound healing [25,26,27,28]. Since electrical signaling is an active response to injury and it is known that the application of external electrical stimulation modulates the rate of corneal epithelial function, especially at the beginning of the wound healing process, the present work aims to analyze the gene and protein expression profiles following AC EF stimulation. As it is also known that commonly used DC EFs are the cause of most faults [29], resulting in the buildup of ions near electrodes that can eventually nullify the applied EF [21], a novel in vitro system for electric stimulation with AC voltage [26] was used in the present study to investigate the differential expression of genes and proteins in corneal epithelial cells. 

## 2. Materials and Methods

TPP tissue culture flasks, dishes, and 6- and 12-well plates were obtained from Sigma-Aldrich (Munich, Germany). The QuantiTect Reverse Transcription kit was purchased from Qiagen (Hilden, Germany) and the innuMIX qPCR MasterMix SyGreen was from Analytik-Jena (Jena, Germany). Primers were synthesized from Metabion GmbH (Martinsried, Germany).

### 2.1. Cell Culture

Telomerase-immortalized human corneal epithelial cell line [30] (hTCEpi; a kind gift from Prof. J. V. Jester, University of California, Irvine, CA, USA; authenticated and characterized according to ATCC standard protocols) between passages 24 and 30 was used in this present study. The hTCEpi cells were cultured in KGM-Gold™ growth medium (Lonza, Köln, Germany). Cells were sub-cultured on T75 tissue culture flasks (Sigma-Aldrich, Munich, Germany), incubated at 37 °C in 5% CO_2_, and passaged every 5–7 days.

### 2.2. Alternating Current (AC) Electric Field (EF) Stimulation

To study the influence of AC electric fields on hTCEpi cells, an in vitro setup developed by Prof. Bader’s group at the Medical University of Rostock, Rostock, Germany, was used [26]. In this system, the electrode setup was modified to perform the EF stimulations in a chamber of a 6-well plate. The pure titanium cylindrical electrodes measuring 14 mm in length and 5 mm in diameter were segregated by a 5 mm-long insulator made up of polyether ether ketone (PEEK). The electrode holders were also made of PEEK and provided a distance of 3 mm between electrodes and the well bottom, thus facilitating growth of the cultured cells below the electrodes. The lid of 6-well plate had pre-drilled holes for the contact titanium rods measuring 35 mm in length. The electrical stimulation was started 14 h after cell seeding. Prior to EF stimulation, the medium was replaced by 4 mL fresh cell culture medium (4.2 mm medium height) without the addition of growth factors. AC EFs were applied continuously for 24 h as a sinusoidal signal of 200 mV and with a frequency of 20 Hz, resulting in a maximum electrical field of 1.4 V/m, as previously described, using a Metrix GX 310 function generator (Metrix Electronics, Hampshire, UK). During stimulation, cells were cultured at 37 °C under 5% CO_2_. For unstimulated control, cells were similarly cultured in a 6-well plate in the presence of the electrode system, however without connecting to the function generator.

### 2.3. Microarray Hybridization

After stimulation with AC EFs for 24 h, hTCEpi cells were lysed in RTL Plus buffer, part of the RNeasy Plus Kit (Qiagen, Hilden, Germany), and RNA was extracted according to the manufacturer’s protocol. The whole RNA samples were quantified spectrophotometrically (NanoDrop, Thermo Fisher, Darmstadt, Germany) and the integrity was controlled using Agilent Bioanalyzer 2100 with the RNA Nano chip kit (Agilent Genomics, Waldbronn, Germany). RNA integrity number values between 9.0 and 9.8 were achieved and 200 ng was used as the starting material. To perform the differential gene expression profiling, Affymetrix Clariom^TM^ S Arrays were used according to the manufacturer’s instructions (Affymetrix, St. Clara, CA, USA). The hybridization was carried out overnight at 45°C in the GeneChip^®^ Hybridization Oven 645 (Affymetrix). The microarray was scanned using the GeneChip Scanner 3000 (Affymetrix) at 0.7 micron resolution.

### 2.4. Microarray Data Analysis

The CEL files generated following the Affymetrix Clariom^TM^ S Arrays were analyzed using R [31]. The CEL files were read using the “oligo” R package. Raw data were normalized using the robust multichip algorithm (RMA) method of “oligo” R package. This normalized data were used to generate heatmap and PCA plots. Differential expression analysis was done using “limma” R package [32]. Differentially expressed genes were defined as genes with adjusted *p*-values < 0.05 and absolute log2 foldchanges > 2. GO and KEGG enrichment analysis was performed using the “clusterProfiler” R package [33]. Transcriptome Analysis Console (TAC) software (v.4.0.1.36) and ShinyGO enrichment tool [34] were also used to analyze and graphical representation the data.

### 2.5. Quantitative Real-Time Polymerase Chain Reaction (qRT-PCR)

Total RNA was isolated from cultured hTCEpi cells after 24 h treatment with AC EFs using the NucleoSpin^®^ RNA kit (Macherey-Nagel, Berlin, Germany) according to the manufacturer’s instructions. Single-stranded cDNA was prepared with 1 µg of total RNA using the QuantiTect Reverse Transcription kit (Qiagen, Hilden, Germany). Intron–exon spanning primers were designed from the respective GenBank sequences using VectorNTI software (ThermoFisher Scientific) based on the minimal hairpin, duplex formation, and guanine cytosine composition. A list of primers used in this study is given in Table 1.

The synthesized cDNA was then used as a template for qRT-PCR using the innuMIX qPCR MasterMix SyGreen and qTower 2.0 (Analytik Jena, Jena, Germany). The cycling conditions used for amplification were 95 °C for 2 min, 40 cycles of 95 °C for 5 s, and 65 °C for 25 s. Later, the expression of all genes was normalized to the expression of the corresponding housekeeping gene, GAPDH. The relative amounts of target mRNA in the unstimulated control cells and EF-treated cells were analyzed using the ΔΔ*C*t method, as described previously [35].

### 2.6. Antibody MicroarrayAanalysis

To analyze the differential expression of various proteins in hTCEpi cells following electric stimulation, cells were treated with AC EFs for 24 h as described above. Later, the cells were collected, washed and frozen cell pellets were sent to Sciomics GmbH (Heidelberg, Germany) for further analysis using scioDiscover protein arrays. For each condition, the array was performed in triplicate. Briefly, proteins were extracted, quantified, and labeled with fluorescent dyes. All six samples were analyzed in a dual-color approach using a reference-based design on scioCD antibody microarrays (Sciomics) targeting 1300 proteins. Each antibody is represented in eight replicates on the array. The arrays were blocked with scioBlock (Sciomics) on a Hybstation 4800 instrument (Tecan, Grödig, Austria). The resulting data were analyzed using the linear models for the microarray data (LIMMA) package of R-Bioconductor after uploading the median signal intensities for differential protein expression. For normalization, a specialized invariant Lowess method was applied, and for analysis of the samples, a one-factorial linear model was fitted with LIMMA, resulting in a two-sided *t*-test or F-test based on moderated statistics. All presented *p* values were adjusted for multiple testing by controlling the false discovery rate according to Benjamini and Hochberg. Proteins were defined as differential for |logFC| > 0.2 and an adjusted *p*-value of < 0.05. Differences in protein abundance between different samples or sample groups are presented as log-fold changes (logFC) calculated for the basis of 2. In a study comparing samples versus control, a logFC = 1 means that the sample group had on average a 2^1^ = 2-fold higher signal as the control group; logFC = −1 stands for 2^−1^ = 1/2 of the signal in the sample as compared to the control group.

## 3. Results

### 3.1. Stimulation of Telomerase-Immortalized Human Corneal Epithelial (hTCEpi) Cells with Alternate Current (AC) Electric Fields (EFs)

**Figure 1:**
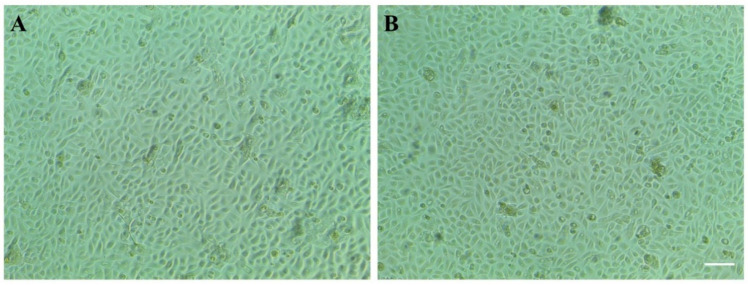
Morphology of hTCEpi cells (**A**) before and (**B**) after AC EF stimulation for 24 h. No significant morphological differences were observed after EF stimulation. Scale bar: 50 µm.

No differences in the morphology of the telomerase-immortalized human corneal epithelial cell line (hTCEpi) were observed during stimulation with AC EFs for 24 h. The distribution of the applied EF strength during AC EF stimulation is shown in Supplementary Figure S1. In addition, EF-stimulated cells maintained a healthy spindle-shaped epithelial morphology and showed growth rate characteristics similar to untreated control cells. Further, no deleterious effects on epithelial cells were observed during AC EF stimulations (Figure 1).

### 3.2. Gene Expession Profiling (GEP) of AC EF-Stimulated hTCEpi Cells

**Figure 2:**
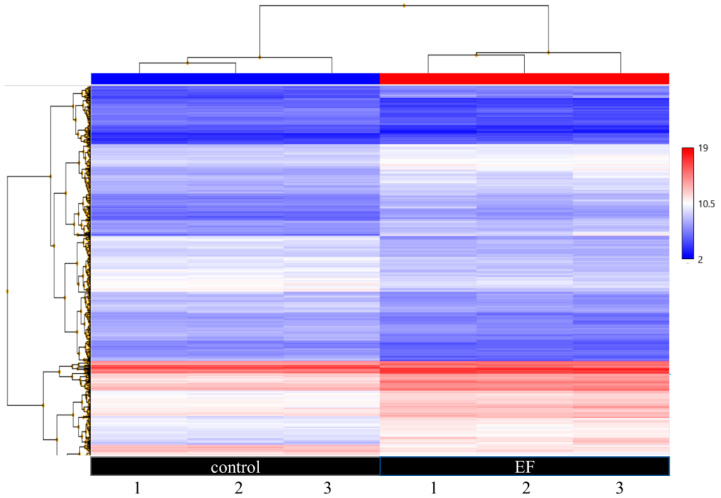
Hierarchical illustration of differentially expressed genes in hTCEpi cells after treatment with AC EFs for 24 h. Both two-fold upregulated and downregulated genes were clustered. Untreated hTCEpi cells served as control. After 24 h of treatment with AC EFs, RNA was isolated and transcriptome-wide gene level expression profiling was performed using Clariom^TM^ S arrays. Affymetrix^®^ Transcription Analysis Console^TM^ (TAC) software (v.4.0.1.36) was used for array scanning, data analysis, signal estimation, and quality control functionality for the expression arrays, which also allow statistical analysis and provide a list of differentially expressed genes. Expression level analysis was performed using the normalization method based on the processing algorithm called the robust multi-array average (RMA), which was improved by Signal Space Transformation (SST-RMA, Affymetrix). Each array was performed in triplicate. Blue indicates a relatively low expression, whereas red indicates a relatively high expression.

Since there were no observable morphological differences in hTCEpi cells following EF stimulation, we further proceeded to study the effects of EF stimulation using gene expression analysis. After AC EF treatment of hTCEpi cells for 24 h, cells were washed once with PBS and collected to isolate RNA. Gene level differential expression analysis was performed using Clariom™ S arrays. Principal component analysis of the RMA normalized microarray data is shown in Supplementary Figure S2A. A clustering heat map of the calibrated samples is shown in Supplementary Figure S3A, whereas a volcano plot of the differentially expressed genes is depicted in Supplementary Figure S3B. Of the 21,448 genes analyzed, only 5.35% of the genes were found to be differentially expressed (556 genes were upregulated and 591 genes were downregulated) after stimulation of hTCEpi cells with AC EFs compared to the control untreated cells (Figure 2).

Gene Ontology analysis using the STRING database of the 1147 genes that are upregulated and downregulated upon stimulation with AC EFs revealed activation of biological processes that are predominantly responsive to external, chemical, and cytokine stimuli, which in turn subsequently leading to the activation of various pathways, which are shown in Table 2. A list of genes that are upregulated and downregulated after treatment with AC EFs is shown in Supplementary Table S1.

### 3.3. Validation of Trancriptome Analysis Data by qRT-PCR

**Figure 3:**
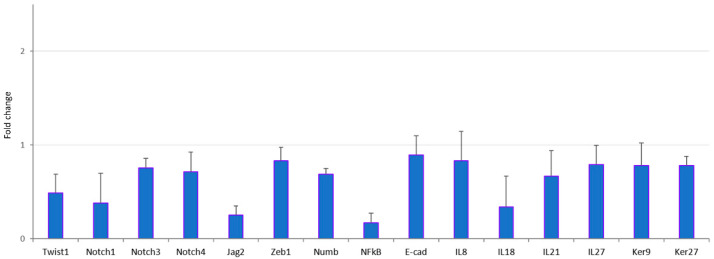
Validation of the Clariom^TM^ S array analyzed transcripts using qRT-PCR. The hTCEpi cells were cultured in the presence of AC EFs and RNA was isolated after 24 h. Reverse transcription was performed using 1 µg of total RNA to prepare cDNA and qRT-PCR was performed to compare the expression analysis of different transcripts using respective primers for Twist1, Notch1, Notch3, Notch4, Jag2, Zeb1, Numb, NFkB, E-Cad, IL8, IL18, IL21, IL27, Ker9 and Ker27. Relative expression patterns were analyzed using the comparative threshold cycle (2^−ΔΔ*C*t^) method. Expression values are represented as fold changes over the control on an arbitrary scale after normalization with GAPDH. Further, these transcripts displayed similar expression profile during Clariom^TM^ S Arrays. Data represent means ± standard deviations of three independent experiments.

To further validate the transcriptome analysis data obtained after treatment of hTCEpi cells with AC EFs, we performed qRT-PCR of various transcripts using RNA isolated from stimulated cells. All the analyzed transcripts showed a similar expression profile as was observed in the gene array analysis (Figure 3).

### 3.4. Antibody Microarray Analysis of Differentially Expressed Proteins in hTCEpi Cells

**Figure 4:**
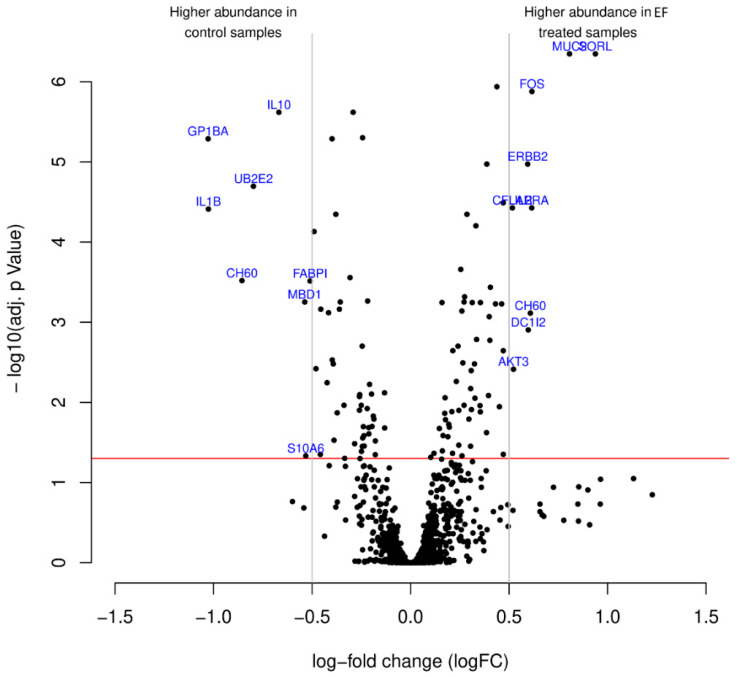
The antibody microarray identified differentially enriched proteins after the treatment of hTCEpi cells with AC EFs. The volcano plot visualizes the *p*-values (adjusted for multiple testing) and corresponding log-fold changes. The log-fold change of the difference in abundance is shown horizontally, whereas the vertical axis represents the significance level. The logFC cutoffs are depicted as vertical lines. The black dots represent analyzed proteins. The horizontal red line indicates an adjusted *p*-value of 0.05, above which all proteins are considered to vary significantly. Proteins with a positive log-fold change display higher abundance in EF-treated samples, whereas proteins with a negative value display higher abundance in control samples.

**Figure 5:**
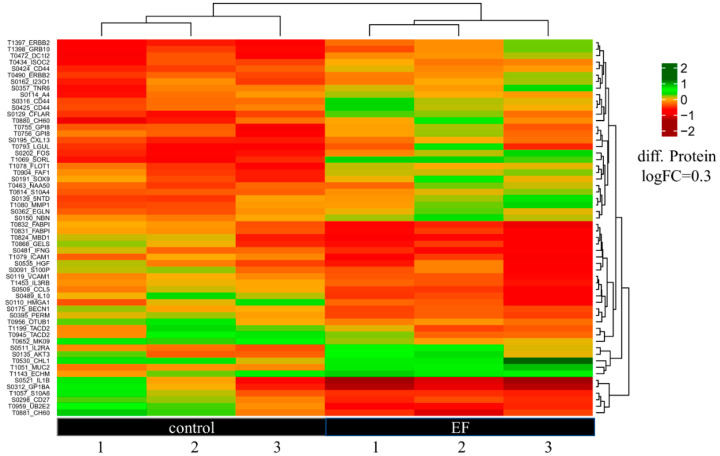
Heatmap of differentially expressed proteins in hTCEpi cells after treatment with AC EFs for 24 h. Both upregulated and downregulated proteins with logFC values of 0.3 were clustered. Untreated hTCEpi cells served as control.

Antibody microarrays were performed using scioDiscover Protein Expression Array (Sciomics GmbH, Germany) to identify putative alterations in hTCEpi cells at the protein level after treatment with AC EFs for 24 h (Figure 4). Regarding the antibody array, hierarchical clustering of the protein extracts based on differentially expressed proteins is shown in Supplementary Figure S2B. Identification of the functional associations between proteins on a genome-wide scale and construction of a protein–protein interaction (PPI) networks for analysis was performed using the STRING database (Available online: https://string-db.org/ (accessed on 14 December 2020)). Analysis of the protein array data revealed that the treatment of cells with AC EFs altered the expression of various protein products and the resulting heatmap of the protein differential expression is depicted in Figure 5. A list of proteins that are upregulated and downregulated after treatment with AC EFs is shown in Supplementary Table S2. The PPI network consisted of 173 nodes interacting through 1261 edges. Of all the differentially abundant proteins, 132 proteins displayed positive logFC values > 0.2 in AC EF-stimulated cells, whereas 63 proteins showed logFC values > 0.2 in untreated control cells.

Analysis of the abundant proteins using the STRING database revealed activation of various diverse cellular signaling pathways in which TNF, MAPK, IL17, and PI3K-Akt signaling pathways were significantly impacted (Table 3).

**Figure 6:**
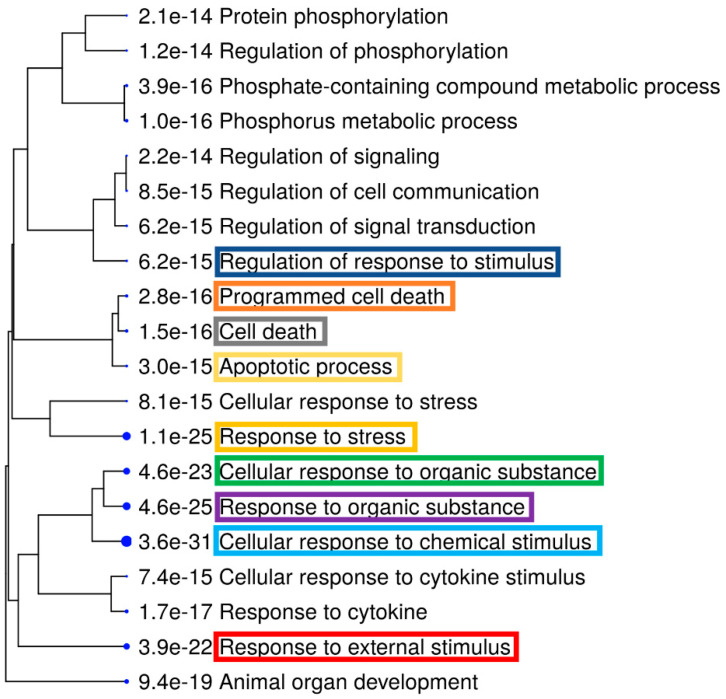
Enriched biological processes after treatment of hTCEpi cells with AC EFs for 24 h. Significantly enriched biological processes following analysis of the gene array data are highlighted as an interactive hierarchical clustering tree using ShinyGO. Biological processes with many shared genes are clustered together. Bigger dots indicate more significant *p*-values. Enriched biological processes that were similar after analysis of both gene and proteins array data are marked and highlighted. Analysis was performed using ShinyGO (v.0.61) (Available online: http://bioinformatics.sdstate.edu/go/ (accessed on 6 January 2021)).

**Figure 7:**
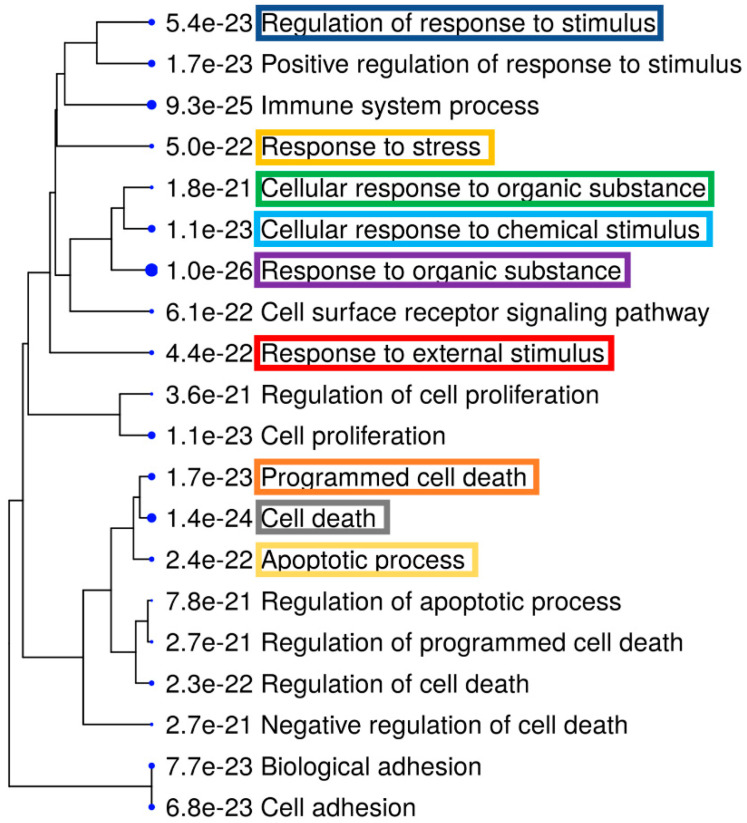
Enriched biological processes after treatment of hTCEpi cells with AC EFs for 24 h. Significantly enriched biological processes following analysis of the antibody array data are highlighted as an interactive hierarchical clustering tree using ShinyGO. Biological processes with many shared genes are clustered together. Bigger dots indicate more significant *p*-values. Enriched biological processes that were similar after analysis of both gene and protein array data are marked and highlighted. Analysis was performed using ShinyGO (v.0.61) (Available online: http://bioinformatics.sdstate.edu/go/ (accessed 6 January 2021)).

**Figure 8:**
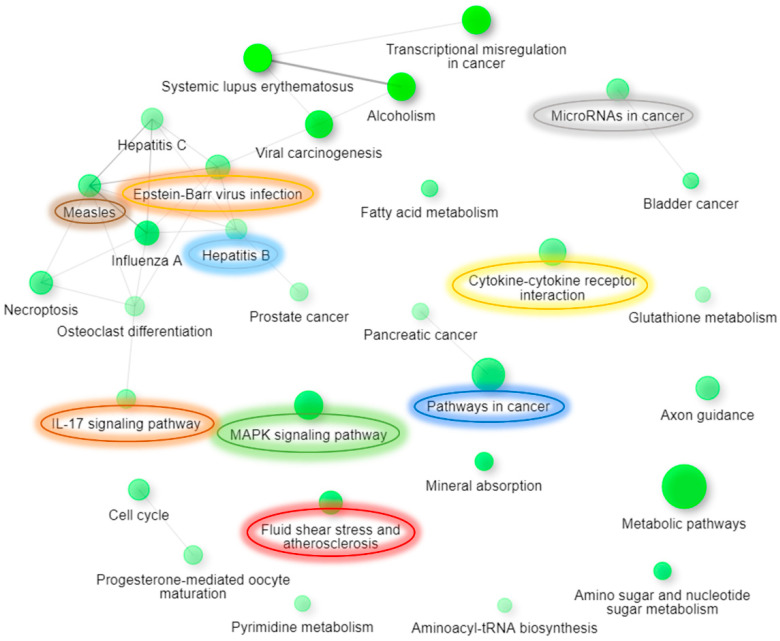
Enriched molecular pathways after treatment of hTCEpi cells with AC EFs for 24 h. The relationships between pathways are depicted and highlighted as an interactive graphical network using ShinyGO. Pathways enriched following analysis of the gene array data are shown. Two pathways (nodes) are connected after sharing 20% or more genes. Enriched pathways that were similar after analysis of both gene and protein array data are marked and highlighted. Node colors illustrate enrichment, whereby darker nodes represent significantly enriched gene sets, while bigger nodes represent larger gene sets. Thicker edges represent more overlapped genes. Analysis was performed using ShinyGO (v.0.61) (Available online: http://bioinformatics.sdstate.edu/go/ (accessed on 6 January 2021)). Edge cut-off = 0.2 and FDR *p*-value cut-off = 0.05.

**Figure 9:**
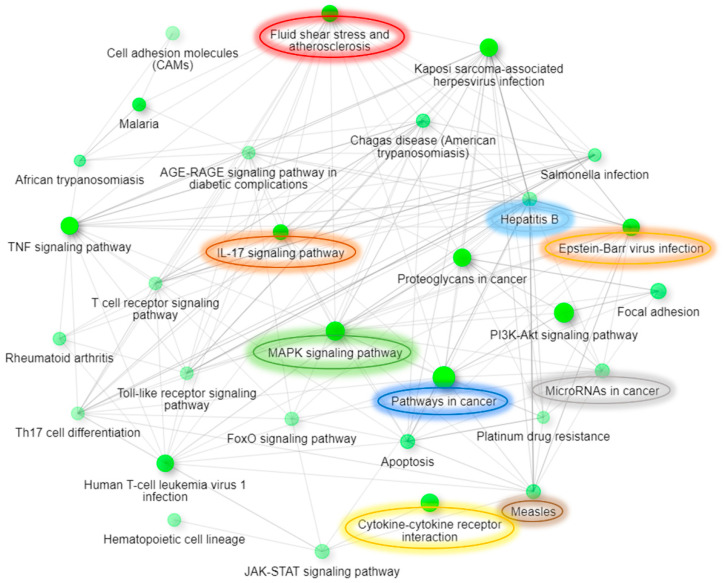
Enriched molecular pathways after treatment of hTCEpi cells with AC EFs for 24 h. The relationships between pathways are depicted and highlighted as an interactive graphical network using ShinyGO. Pathways enriched following analysis of the antibody array data are shown. Two pathways (nodes) are connected after sharing 20% or more genes. Enriched pathways that were similar after analysis of both gene and protein array data are marked and highlighted. Node colors illustrate enrichment, whereby darker nodes represent significantly enriched gene sets, while bigger nodes represent larger gene sets. Thicker edges represent more overlapped genes. Analysis was performed using ShinyGO (v.0.61) (Available online: http://bioinformatics.sdstate.edu/go/ (accessed on 6 January 2021)). Edge cut-off = 0.2 and FDR *p*-value cut-off = 0.05.

Further, we also performed ShinyGO [34] Gene Ontology enrichment analysis using differentially expressed up- and downregulated genes and proteins based on the data from both arrays. Graphical representations of enriched biological processes and pathways are shown in Figure 6, Figure 7, Figure 8 and Figure 9. We observed enrichment of 9 similar biological processes and 9 similar pathways in both arrays, confirming activation of similar cellular events at both the gene and protein levels following AC EF stimulation. Further, in comparison to the gene array data, protein array data showed enrichment of more activated pathways through several interconnecting networks.

## 4. Discussion and Conclusions

In cells, intrinsic endogenous DC EFs serve as morphogenetic cues and are necessary for several important cellular responses, including activation of multiple signaling pathways, cell migration, nerve regeneration, and wound healing [16,36,37]. Further, DC EFs are known to induce asymmetry in the distribution, arrangement of membrane receptors, and activate intracellular signaling cascades in the cathode-facing side, resulting in the growth of neurites in the cathode direction [20,38,39]. In contrast, in vitro stimulation with small AC EFs has no effect on neural progenitor cell alignment, viability, differentiation, or proliferation, similar to control unstimulated cells [18]. Additionally, an increase in AC EF frequency influences neural stem cell viability and differentiation by slowing down their maturation [40], suggesting a lineage control role of AC EFs on neural cells [20]. Based on these observations and due to the presence of the highest number of free nerve endings in the cornea, along with the existence of stem cells in the corneal limbus, it is also necessary to study the consequences of the application of AC EFs on corneal cells. Since epithelial cells are mainly accountable for the corneal wound healing process and as the effects of external AC EFs in modulating the epithelial cell function is presently not known, this study was carried out to understand the influence of external AC EF stimulation on corneal epithelial cell function. 

Upon stimulation with AC EFs, similar to PC12 cells [41], no significant differences in corneal epithelial cell viability or proliferation were observed. All of the literature reports so far used constant electric stimulation to study the effects of external EF applications on corneal epithelial cells and showed increased cell division, directional migration [42], regeneration [43], and wound healing [1]. MMPs play a crucial role in corneal wound healing [44] and corneal epithelial cells show enhanced MMP3 and unaltered MMP2 and MMP9 expression following DC EF stimulation [45]. Our gene array data also showed increased expression of MMP1 (48-fold), MMP10 (20-fold), and MMP9 (4-fold) and decreased expression of MMP2, MMP13, and MMP19 (2-fold each), whereas protein array data showed upregulation of MMPs 1, 7, 10, and 12. The application of DC EFs is known to significantly increase the activation of AKT and impact the PI3K/AKT pathway, which plays an important role in cell survival, growth, migration, and metabolism [46]. Further, DC-induced activation of the PI3K/AKT pathway was also reported in different cell types, including neural progenitor cells [47], Schwann cells [48], and human umbilical vein endothelial cells (HUVEC) [49], as well as in ocular cells, including the corneal epithelium [6,50]. Activation of the MAPK signaling pathway is also reported through the application of EFs in neuronal cells [51,52], fibroblasts, HUVEC [53], and corneal epithelial cells [6]. Likewise, we also observed activation of PI3K/AKT and MAPK signaling pathways, along with other interconnected pathways, including T cell receptor signaling, Toll-like receptor signaling, TNF signaling, IL-17 signaling, FoxO signaling, and focal adhesion pathways, upon stimulation of corneal epithelial cells with oscillating EFs. In addition, electric stimulation upregulates expression of RAGs, increases neurotrophin signaling, accelerates axon guidance and outgrowth during peripheral nerve regeneration [25,54,55,56,57], and is also known to activate axon guidance, along with various metabolic and signaling pathways that play crucial interconnecting roles during axon regeneration [17,57,58,59]. Accordingly, our gene array data analysis also showed activation of similar pathways during AC EF stimulation of corneal epithelial cells. 

Generally, it is well known that following cell-specific stimulation, the resulting gene expression profiles measured using gene arrays are inadequate to predict protein expression and subsequent functional outcomes on a genomic scale, since interrelationships between mRNA and protein levels vary greatly due to post-transcriptional mechanisms, as well as post-translational modifications. Influencing factors such as the mRNA transcription rate, stability, translational regulation, along with protein activity and degradation serve as limiting factors and controlling elements in a given cell at the physiological level when interpreting the after effects of that cell-specific stimulus [60,61,62]. However, in our study, comparison of the expression profiles of certain genes and their corresponding protein abundance levels showed similar patterns following stimulation with AC EFs. Accordingly, enriched pathways and processes following analysis of the gene and antibody array data using the STRING database also showed consistent enrichment of similar biological processes and signaling pathways, further highlighting the balanced, steady state of the epithelial cells during AC EF stimulation [62]. Additionally, antibody array data showed enrichment of various interconnecting signaling events due to the involvement of similar molecules in multiple pathways, which was not observed during gene array data analysis. Since electric stimulation is considered as a master regulator of regeneration-associated cellular networks [27,63], stimulation with AC EFs also switches on several intracellular pathways, and thus can be implemented, upon further testing and validation at shorter time points, as a potential therapeutic approach for corneal tissue regeneration. 

## Figures and Tables

**Table 1:** List of primers used in the present study.

Target	Primer	Primer Sequence (5′-3′)	GenBank Accession
Twist1	Fwd	CAGCCTGAGCAACAGCGAGGA	NM_000474
	Rev	CGAACGCCTCGTTCAGCGAC	
Notch1	Fwd	GCAGTGCTTCCAGAGTGCCACC	NM_017617
	Rev	AACCGGAACTTCTTGGTCTCCAGG	
Notch3	Fwd	GGCCAGGCCATGGTCTTCC	NM_000435
	Rev	TCAGGGAACCAGAGGGTGCTG	
Notch4	Fwd	AGGCTGGAGCCAACCCCAAC	NM_004557
	Rev	CAGCCCCTTCCAGCAGCGT	
Jag2	Fwd	CCTGATTGGCGGCTATTACTGTGA	NM_002226
	Rev	CCCGCAGCCATCGATCACTC	
Zeb1	Fwd	GCAGGGCACACCAGAAGCCA	NM_001128128
	Rev	GTGAGCTATAGGAGCCAGAATGGGAA	
Numb	Fwd	CAAGCTAATGGCACTGACTCAGCC	NM_001005743
	Rev	CTGAGGGTGGCCTGCAGTGC	
NFkB	Fwd	GCACCTAGCTGCCAAAGAAGGAC	NM_001165412
	Rev	GCTCCTGCTGCTTTGAGAAGAGC	
E-cadherin	Fwd	GCTTGGATTTTGAGGCCAAGCA	NM_004360
	Rev	AATCTCCAGCCAGTTGGCAGTGTC	
IL8	Fwd	CGTGGCTCTCTTGGCAGCC	NM_000584
	Rev	TGTGTTGGCGCAGTGTGGTC	
IL18	Fwd	CCAAGGAAATCGGCCTCTATTTG	NM_001562
	Rev	TCAGGAGGATTCATTTCCTTAAAGGA	
IL21	Fwd	GCCACATGATTAGAATGCGTCAAC	NM_021803
	Rev	TGTATTTGCTGACTTTAGTTGGGCC	
IL27	Fwd	CGGAGCGTCTCTGCTTCATCTC	NM_145659
	Rev	AGGTTGAATCCTGCAGCCAGC	
Ker9	Fwd	CTCTCCTGGACATTGACAACACTCGC	NM_000226
	Rev	AGCACCTGCCGCAGGCCAT	
Ker27	Fwd	GCGAGCTGAGTACGAAGCCCTCG	NM_181537
	Rev	CTCATTCCGGGCTGAGGTGGTG	
GAPDH	Fwd	GGATATTGTTGCCATCAATGACCC	NM_002046
	Rev	TCTCGCTCCTGGAAGATGGTGA	

**Table 2:** Significantly impacted pathways in hTCEpi cells after AC EF stimulation based on the Clariom™ S gene array data.

KEGG Pathway	Pathway Description	Observed Gene Count	False Discovery Rate
hsa00520	Amino sugar and nucleotide sugar metabolism	13 of 48	0.0036
hsa04010	MAPK signaling pathway	35 of 293	0.0036
hsa04978	Mineral absorption	13 of 51	0.0036
hsa05162	Measles	21 of 133	0.0036
hsa05164	Influenza A	25 of 168	0.0036
hsa05202	Transcriptional misregulation in cancer	25 of 169	0.0036
hsa05418	Fluid shear stress and atherosclerosis	22 of 133	0.0036
hsa01100	Metabolic pathways	100 of 1250	0.0074
hsa01212	Fatty acid metabolism	11 of 48	0.0074
hsa05160	Hepatitis C	19 of 131	0.0074
hsa05200	Pathways in cancer	49 of 515	0.0074
hsa05203	Viral carcinogenesis	24 of 183	0.0074
hsa05206	MicroRNAs in cancer	21 of 149	0.0074
hsa05219	Bladder cancer	10 of 41	0.0074
hsa04110	Cell cycle	18 of 123	0.0076
hsa05168	Herpes simplex infection	23 of 181	0.0076
hsa04360	Axon guidance	22 of 173	0.0093
hsa04657	IL-17 signaling pathway	14 of 92	0.0194
hsa04060	Cytokine-cytokine receptor interaction	28 of 263	0.0206
hsa04914	Progesterone-mediated oocyte maturation	14 of 94	0.021
hsa00970	Aminoacyl-tRNA biosynthesis	9 of 44	0.0213
hsa05161	Hepatitis B	18 of 142	0.0229
hsa01040	Biosynthesis of unsaturated fatty acids	6 of 23	0.0426
hsa05322	Systemic lupus erythematosus	13 of 94	0.0454
hsa04217	Necroptosis	18 of 155	0.0474

Analysis of the gene array data was performed using TAC software (v.4.0.1.36) and putative differentially expressed up- and downregulated gene function was analyzed to identify impacted signaling pathways using the STRING database, version 11 (Available online: http://string-db.org (accessed on 14 December 2020)). KEGG = Kyoto Encyclopedia of Genes and Genomes.

**Table 3:** Significantly impacted signaling pathways in hTCEpi cells after AC EF stimulation based on the antibody microarray data.

KEGG Pathway	Pathway Description	Protein Count	False Discovery Rate	Matching Proteins in Network
hsa04668	TNF signaling pathway	17	1.95 × 10^−13^	MAPK12, MAPK1, AKT3, ICAM1, VCAM1, IL15, CSF2, FOS, CASP3, CFLAR, SELE, FAS, MAPK9, TNF, MAP2K4, PGAM5, CCL5
hsa05200	Pathways in cancer	27	2.95 × 10^−11^	MAPK1, HGF, IFNG, CCND2, AKT3, IL7, FGF2, STAT3, TP53, ERBB2, IL15, HSP90B1, IL7R, FOS, CASP3, GSTM1, KLK3, MET, MMP1, CASP9, FAS, RAC1, IL2RA, ETS1, JAK3, MAPK9, CUL2
hsa05418	Fluid shear stress and atherosclerosis	16	2.95 × 10^−11^	MAPK12, IFNG, AKT3, KDR, ICAM1, TP53, VCAM1, HSP90B1, FOS, GSTM1, SELE, CAV3, RAC1, MAPK9, TNF, MAP2K4
hsa04010	MAPK signaling pathway	21	3.06 × 10^−11^	MAPK12, MAPK1, HGF, HSPB1, AKT3, KDR, FGF2, TP53, ERBB2, CD14, FOS, CASP3, MET, FAS, RAC1, HSPA1A, MAPK9, TNF, MAP2K4, DDIT3, NTF4
hsa04060	Cytokine-cytokine receptor interaction	20	3.06 × 10^−11^	HGF, IFNG, TNFSF10, CCR7, IL7, KDR, CXCL13, IL15, CSF2, CCL11, IL7R, MET, FAS, IL2RA, CCL8, TNF, IL10, TNFSF14, CCL5, TNFRSF9
hsa04657	IL-17 signaling pathway	13	2.29 × 10^−10^	MAPK12, MAPK1, IFNG, CSF2, HSP90B1, CCL11, FOS, CASP3, MMP1, MAPK4, MAPK9, TNF, MUC5B
hsa04151	PI3K-Akt signaling pathway	21	3.07 × 10^−10^	MAPK1, HGF, VTN, CCND2, AKT3, IL7, KDR, FGF2, TP53, ERBB2, ITGA5, HSP90B1, IL7R, MET, CASP9, RAC1, THBS3, IL2RA, JAK3, PPP2R5D, NTF4
hsa04933	AGE-RAGE signaling pathway in diabetic complications	11	4.79 × 10^−8^	MAPK12, MAPK1, AKT3, STAT3, ICAM1, VCAM1, CASP3, SELE, RAC1, MAPK9, TNF
hsa04660	T cell receptor signaling pathway	11	4.95 × 10^−8^	MAPK12, MAPK1, IFNG, AKT3, CSF2, FOS, CD28, MAPK9, TNF, PTPRC, IL10
hsa04620	Toll-like receptor signaling pathway	11	6.21 × 10^−8^	MAPK12, MAPK1, AKT3, CD14, FOS, CD86, RAC1, MAPK9, TNF, MAP2K4, CCL5
hsa04510	Focal adhesion	14	6.84 × 10^−8^	MAPK1, HGF, VTN, CCND2, AKT3, KDR, ERBB2, ITGA5, MET, CAV3, RAC1, THBS3, ACTN1, MAPK9
hsa05164	Influenza A	13	8.98 × 10^−8^	MAPK12, MAPK1, IFNG, TNFSF10, AKT3, ICAM1, CASP9, FAS, HSPA1A, MAPK9, TNF, MAP2K4, CCL5
hsa04630	Jak-STAT signaling pathway	12	3.49 × 10^−7^	IFNG, CCND2, AKT3, IL7, STAT3, IL15, CSF2, IL7R, AOX1, IL2RA, JAK3, IL10
hsa04068	FoxO signaling pathway	11	3.99 × 10^−7^	MAPK12, MAPK1, TNFSF10, CCND2, AKT3, STAT3, ATM, IL7R, BNIP3, MAPK9, IL10
hsa05162	Measles	11	4.63 × 10^−7^	IFNG, TNFSF10, CCND2, AKT3, STAT3, TP53, CD28, FAS, HSPA1A, IL2RA, JAK3
hsa05161	Hepatitis B	11	8.35 × 10^−7^	MAPK1, AKT3, STAT3, TP53, FOS, CASP3, CASP9, FAS, MAPK9, TNF, MAP2K4
hsa04664	Fc epsilon RI signaling pathway	8	2.19 × 10^−6^	MAPK12, MAPK1, AKT3, CSF2, RAC1, MAPK9, TNF, MAP2K4
hsa05168	Herpes simplex infection	11	6.12 × 10^−6^	CDC34, IFNG, TP53, IL15, FOS, CASP3, FAS, MAPK9, TNF, TNFSF1, CCL5
hsa04514	Cell adhesion molecules (CAMs)	10	4.92 × 10^−6^	SELP, ICAM1, VCAM1, CD28, SELE, CD86, VTCN1, CD99, PDCD1LG2, PTPRC
hsa04370	VEGF signaling pathway	7	9.52 × 10^−6^	MAPK12, MAPK1, HSPB1, AKT3, KDR, CASP9, RAC1
hsa04217	Necroptosis	10	1.05 × 10^−5^	IFNG, TNFSF10, STAT3, CFLAR, FAS, FAF1, JAK3, MAPK9, TNF, PGAM5
hsa04115	p53 signaling pathway	7	1.92 × 10^−5^	CCND2, TP53, ATM, CASP3, CASP9, FAS, CHEK2
hsa04917	Prolactin signaling pathway	7	2.02 × 10^−5^	MAPK12, MAPK1, CCND2, AKT3, STAT3, FOS, MAPK9
hsa04062	Chemokine signaling pathway	10	3.16 × 10^−5^	MAPK1, CCR7, AKT3, STAT3, CXCL13, CCL11, RAC1, CCL8, JAK3, CCL5
hsa04512	ECM-receptor interaction	7	5.07 × 10^−5^	VTN, ITGA5, GP1BA, CD47, THBS3, HMMR, CD44
hsa04071	Sphingolipid signaling pathway	8	5.57 × 10^−5^	MAPK12, MAPK1, AKT3, TP53, RAC1, MAPK9, TNF, PPP2R5D
hsa04064	NF-kappa B signaling pathway	7	0.00011	ICAM1, ATM, VCAM1, CD14, CFLAR, TNF, TNFSF14
hsa04066	HIF-1 signaling pathway	7	0.00013	MAPK1, IFNG, ENO1, AKT3, STAT3, ERBB2, CUL2
hsa04014	Ras signaling pathway	10	0.00016	MAPK1, HGF, AKT3, KDR, FGF2, MET, RAC1, ETS1, MAPK9, NTF4
hsa04722	Neurotrophin signaling pathway	7	0.00034	MAPK12, MAPK1, AKT3, TP53, RAC1, MAPK9, NTF4
hsa04926	Relaxin signaling pathway	7	0.00066	MAPK12, MAPK1, AKT3, FOS, MMP1, MAPK9, MAP2K4
hsa04015	Rap1 signaling pathway	8	0.0015	MAPK12, MAPK1, HGF, AKT3, KDR, FGF2, MET, RAC1
hsa05219	Bladder cancer	4	0.0017	MAPK1, TP53, ERBB2, MMP1
hsa04110	Cell cycle	6	0.0025	CCND2, TP53, ATM, CDC25C, CHEK2, CDKN1C
hsa04012	ErbB signaling pathway	5	0.0027	MAPK1, AKT3, ERBB2, MAPK9, MAP2K4
hsa04550	Signaling pathways regulating pluripotency of stem cells	6	0.0042	MAPK12, MAPK1, AKT3, FGF2, STAT3, JAK3
hsa04920	Adipocytokine signaling pathway	4	0.0089	AKT3, STAT3, MAPK9, TNF
hsa04919	Thyroid hormone signaling pathway	5	0.0094	MAPK1, AKT3, NCOR1, TP53, CASP9
hsa04662	B cell receptor signaling pathway	4	0.0096	MAPK1, AKT3, FOS, RAC1
hsa04915	Estrogen signaling pathway	5	0.0154	MAPK1, AKT3, HSP90B1, FOS, HSPA1A
hsa04024	cAMP signaling pathway	6	0.0179	MAPK1, SOX9, AKT3, FOS, RAC1, MAPK9
hsa04912	GnRH signaling pathway	4	0.0179	MAPK12, MAPK1, MAPK9, MAP2K4
hsa04310	Wnt signaling pathway	5	0.0197	MMP7, CCND2, TP53, RAC1, MAPK9
hsa04810	Regulation of actin cytoskeleton	6	0.0213	MAPK1, FGF2, ITGA5, CD14, RAC1, ACTN1
hsa04141	Protein processing in endoplasmic reticulum	5	0.0298	AMFR, HSP90B1, HSPA1A, MAPK9, DDIT3
hsa04621	NOD-like receptor signaling pathway	5	0.0329	MAPK12, MAPK1, MAPK9, TNF, CCL5
hsa04622	RIG-I-like receptor signaling pathway	3	0.0468	MAPK12, MAPK9, TNF
hsa03320	PPAR signaling pathway	3	0.0497	FABP2, FABP5, MMP1

After antibody microarray, the functions of highly enriched proteins and their interactions with other proteins were analyzed to identify impacted signaling pathways using the STRING database, version 11 (Available online: http://string-db.org (accessed on 14 December 2020)). KEGG = Kyoto Encyclopedia of Genes and Genomes.

## Data Availability

Data is contained within the supplementary material.

## Supplementary Materials

The following are available online at https://www.mdpi.com/2073-4425/12/2/299/s1: Figure S1: Distribution of applied AC EF strength in a 6-well plate during hTCEpi cell stimulation (electric potential: 200 mV; frequency: 20 Hz; wave form). Figure S2: Analysis of the gene and protein array data: (A) principal component analysis of the RMA-normalized microarray data; (B) hierarchical clustering of the protein extracts based on differentially expressed proteins. Figure S3: Analysis of the gene array data: (A) clustering heat map of the calibrated samples; (B) volcano plot of the differentially expressed genes. Table S1: Gene array data. Table S2: Antibody array data.
