# Supplementary material for: Analysis of the Differential Gene and Protein Expression Profiles of Corneal Epithelial Cells Stimulated with Alternating Current Electric Fields
Source: Genes (Basel). 2021 Feb 20;12(2):299. doi: 10.3390/genes12020299 (PMC7924190; doi:10.3390/genes12020299)
Supplement: Supplementary file 1 [file genes-12-00299-s001.pdf]

Supplementary information

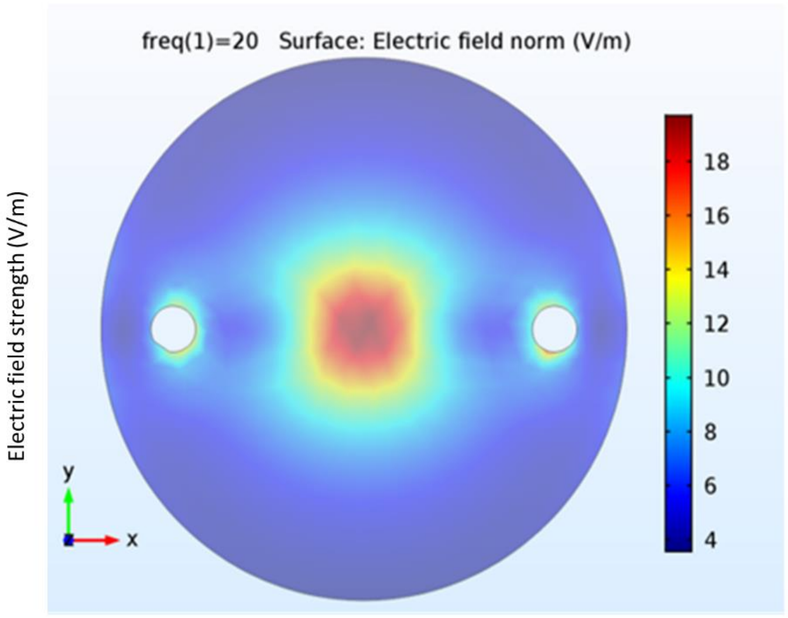

Supplementary Figure S1: Distribution of applied AC EF strength in a 6–well plate during hTCEpi cell stimulation. (electric potential: 200 mV; frequency: 20 Hz; wave form). Simulated by finite element method and the color–coding of the scale bar defines the range of values.

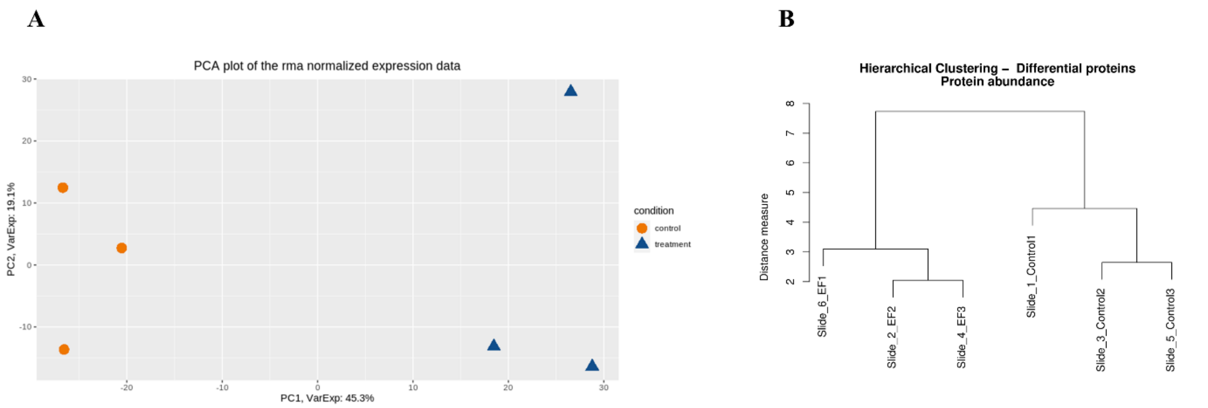

Supplementary Figure S2: Analysis of the gene and protein array data. (A) represents principal component analysis of the RMA normalized microarray data. (B) represents hierarchical clustering of the protein extracts based on differentially expressed proteins.

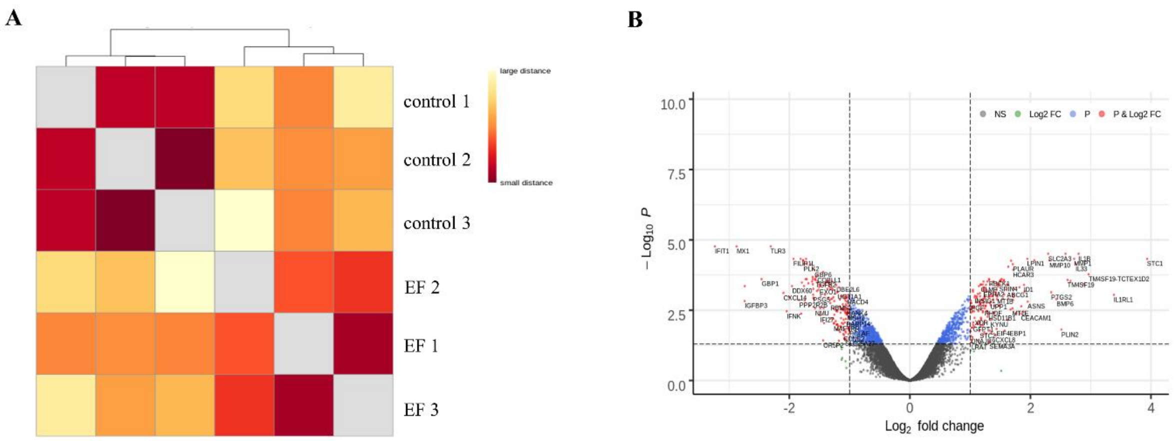

Supplementary Figure S3: Analysis of the gene array data. (A) represents the clustering heat map of the calibrated samples. (B) represents Volcano plot of the differentially expressed genes. X−axis shows the measured fold changes in the expression whereas the y−axis shows the significance of the change in terms of negative log (base 10) of the p−value. The threshold used to select the differentially expressed genes is 2 for expression change and 0.05 for significance.

Supplementary Table S1

<u>Gene array data:</u>

upregulated during AC EF stimulation		downregulated during AC EF stimulation	
Gene Symbol	***Fold Change***	***Gene Symbol***	***Fold Change***
MMP1	48.87	SAMM50	−2
STC1	47.51	PGAP1	−2
PAPL	45.43	PRODH	−2
HBEGF	34.58	CEP97	−2
IL1B	33.7	EML1	−2
TM4SF19	27.27	IRX2	−2
TRIB3	22.69	FETUB	−2.01
MMP10	20.93	METAP1D	−2.01
TM4SF19−TCTEX1D2	20.69	ZMYM3	−2.01
PTGS2	20.3	TGFBR3	−2.01
SLC2A3	19.33	ZMAT3	−2.01
IL1RL1	19.2	FAM111A	−2.01
RAC2	14.32	LRRC1	−2.01
PLAUR	13.05	OR5P3	−2.01
BMP6	12.01	EFNA5	−2.01
ID1	11.59	NCAPG	−2.01
PRDM1	11.1	LDAH	−2.01
SERPINB1	10.96	RFC5	−2.02
IL33	10.62	FAM46B	−2.02
LPIN1; MIR548S	10.61	AGPS	−2.02
MT1E	10.52	USP54	−2.02
CEACAM1	10.35	VKORC1	−2.02

ETV5	10.3	PIGK	−2.02
GK	9.85	TMEM14C	−2.02
MT1B; MT1CP	9.66	GPR137B	−2.02
MT1F	9.06	PCDH18	−2.02
MT1G	8.84	ORC1	−2.02
HCAR3	8.73	LRRC8D	−2.02
PSAT1	8.68	SLURP1	−2.03
PRR9	8.64	PIK3IP1	−2.03
TMEM2	8.64	LIMK2	−2.03
ACSS2	8.58	TTC21B	−2.03
VEGFA	8.55	C10orf99	−2.03
IL24	8.52	GSR	−2.03
HCAR2	8.41	HAUS4; MIR4707	−2.03
SLC2A14	8.01	MSRB1	−2.03
PRSS22	7.93	FYCO1	−2.04
MT1A	7.85	PHF21A	−2.04
PLIN2	7.8	SYT14	−2.04
ANTXR2	7.61	FANCD2	−2.04
TFPI2	7.44	CCNF	−2.04
MT1L	7.43	ECI2	−2.04
CHAC1	7.42	TMEM14B	−2.05
LIPE	7.4	ARL6IP5	−2.05
STK10	7.33	IGSF3	−2.05
MT1X	7.07	TMCO4	−2.05
ABCG1	7.03	COMMD3−BMI1; BMI1	−2.05
HSD17B2	6.98	MTX3	−2.05
ASNS	6.87	DIAPH3	−2.05
TXNRD1	6.86	FBXO5	−2.05
IRAK2	6.83	GOLGA8K; ULK4P1	−2.05
DDIT4	6.81	HIST2H4B; HIST2H4A	−2.06
FAM107B	6.7	C14orf1	−2.06
TSC22D1	6.64	GSTO2	−2.06
MXD1	6.6	ARSI	−2.06
MVD	6.55	LMBRD2	−2.06
GDF15	6.35	AURKA	−2.06
TRIB1	6.3	KIF23	−2.06
MT1H	6.25	ZDHHC24	−2.06
PHLDA1	6.15	TNFAIP8L1	−2.06
CLDN4	6.1	RAD51B	−2.07
OVOL1	6.04	CNTNAP2	−2.07
PNLIPRP3	5.95	PIK3C2B	−2.07
FDPS	5.94	GALNT10	−2.07
FAM84B	5.93	RAB2A	−2.07
MT2A	5.89	ATG4C	−2.07
ITGA2	5.85	PRKCQ	−2.07
SLC7A11	5.81	GSN	−2.07
RBCK1	5.77	GADD45A	−2.07
ACOT7	5.7	CNDP2	−2.07

SPRY4	5.69	ZNF658	−2.08
ATF4	5.68	LAP3	−2.08
FAM83A	5.66	FTO	−2.08
IER3	5.59	C1orf233	−2.08
UPP1	5.56	IGFBP6	−2.09
PITPNC1	5.53	JAG2	−2.09
KYNU	5.51	UQCC2	−2.09
TMPRSS4	5.5	MGST3	−2.09
CXCL8	5.49	DPCD	−2.09
CEMIP	5.39	DSEL	−2.09
SPIN4	5.3	ALDH4A1	−2.09
FOSL1	5.26	KRT9	−2.09
FERMT1	5.21	AIG1	−2.09
EREG	5.07	ASB8	−2.1
SDR9C7	5.04	EBAG9	−2.1
ERN1	5.03	PNPT1	−2.1
C3	5.02	FRMD4A	−2.1
PDE7A	5	SLC39A10	−2.1
PHLDA2	4.99	CEP19	−2.1
ARID3B	4.98	FAM234A; ARHGDIG	−2.1
NT5E	4.93	MAP3K12	−2.1
ARG2	4.93	MCOLN3	−2.1
EPHA2	4.85	CYB5R1	−2.11
LRP8	4.84	INPP5D	−2.11
SDCBP2	4.81	C5orf28	−2.11
HEPHL1	4.76	CLEC7A	−2.12
ARHGEF2	4.76	SUOX	−2.12
RHCG	4.76	PAM	−2.12
GFPT2	4.73	HMGN3	−2.12
CEACAM5	4.65	KIF20A	−2.12
XDH	4.65	CASP14	−2.12
BMP2	4.63	CCNB2	−2.12
SMOC1	4.63	CRISPLD1	−2.12
NAV3	4.63	EPHA4	−2.12
AQP3	4.6	LMNB1	−2.12
HSPB8	4.54	DST	−2.12
GPAT3	4.53	MACC1	−2.13
TMCC3; MIR7844	4.5	DCP1B	−2.13
HAS3	4.44	MMP19	−2.13
SPRY2	4.43	IRF9	−2.13
DUSP1	4.42	PLD1	−2.13
BHLHE40	4.42	CDC7	−2.13
CLMP	4.41	TENM2	−2.13
KCNK1	4.38	ZNFX1	−2.13
TNFRSF10A	4.35	MKI67	−2.13
SOD2	4.29	BACE1	−2.14
DUSP10	4.28	SERPINE1	−2.14
MT1M	4.28	RFX2	−2.14

DAB2	4.27	ZNF385B	−2.14
KDM7A	4.26	NSMCE1	−2.14
ARSJ	4.25	PAN2; CNPY2	−2.14
CNFN	4.2	NAV2	−2.14
SLITRK6	4.2	PDK2	−2.14
DUSP6	4.17	ACKR3	−2.14
CBS	4.17	TNS3	−2.14
OSMR	4.16	ACER1	−2.14
FAM214B	4.15	ASAH1	−2.14
CRY1	4.12	SLC48A1	−2.15
RHOF	4.07	IFIT5	−2.15
EIF4EBP1	4.06	DPYD	−2.15
RORA	4	SRD5A1	−2.15
AKIRIN2	3.98	EFNB2	−2.15
MMP9	3.94	STAT2	−2.15
ITGB8	3.92	KIF20B	−2.15
ITPRIP	3.92	PPIC	−2.16
MT1IP	3.9	HIST1H3C	−2.16
INSIG1	3.88	EGLN3	−2.16
NPC1	3.87	VGLL1	−2.16
DDIT3	3.85	ZNF462	−2.16
PPIF	3.83	PIGC	−2.16
DUSP4	3.83	DHFR; DHFRP1	−2.16
GRPEL2	3.81	VSNL1	−2.17
FHL1	3.79	WDR76	−2.17
SNX9	3.78	PALMD	−2.17
PTPRE	3.77	TPD52L1	−2.17
PLAT	3.76	CDCA3	−2.17
LDLR; MIR6886	3.73	IARS2	−2.17
CRCT1	3.72	LYPLAL1	−2.18
IL1A	3.68	PHKB	−2.18
PNP	3.64	GOLM1	−2.18
GPCPD1	3.64	NPL	−2.18
PLCXD1	3.63	FAM89A; MIR1182	−2.18
NCF2	3.62	SH3D19	−2.18
MTHFD2	3.6	UNC93B1	−2.18
NR4A1	3.57	DHFR	−2.18
GRB7	3.57	RAB30	−2.19
NRIP1	3.55	CLDN1	−2.19
GJB4	3.55	TCTN1	−2.19
TMC7	3.53	CHPT1	−2.19
C20orf197	3.5	KIF18A	−2.2
PCSK9	3.48	THSD1	−2.2
PNPLA3	3.47	TMEM9B	−2.21
ARFGAP3	3.46	OVCH2	−2.21
NET1	3.45	CCDC109B	−2.21
SEMA7A	3.45	GSTK1	−2.21
GARS	3.42	WWOX	−2.21

FAM43A	3.38	DHX58	−2.21
KLK9	3.38	EPHB2	−2.21
TBX3	3.37	TPM2	−2.21
SMTN	3.37	SGK1	−2.21
MBD1	3.35	PDLIM1	−2.22
ADGRE2	3.33	TNFRSF21	−2.22
ZNF460	3.32	EPB41L4B	−2.22
STK17B	3.31	GALNT5	−2.22
SULT2B1	3.29	TK1	−2.23
FLG	3.28	DNAJC6	−2.23
ADAM19	3.25	PORCN	−2.23
CCNA1	3.25	FKBP5	−2.24
FASN	3.25	KRT14	−2.24
UBALD2	3.24	TMEM237	−2.24
KLHL21	3.24	ARRDC3	−2.24
P2RX4	3.23	ENPP1	−2.24
CBS	3.23	PUS10	−2.25
INHBA	3.23	OR5P2	−2.25
FOXD1	3.16	PRODH; LOC102724788;	−2.25
MAOA	3.16	ACADSB	−2.25
PFKFB4; MIR6823	3.16	ZNF138	−2.25
PLAU	3.14	SLC25A27	−2.25
TNFSF9	3.13	ZC3HAV1	−2.25
KIAA0040	3.12	MELK	−2.26
SHMT2	3.12	DUSP14	−2.26
EFTUD1	3.11	DERA	−2.26
TMEM39A	3.09	MAPK10	−2.26
E2F7	3.08	ATRAID	−2.26
LRRC8C	3.07	ZNF626	−2.26
TFRC	3.07	CCPG1; MIR628	−2.26
BCAR1	3.06	TMEM254	−2.26
SLC2A6	3.06	TCTN2	−2.27
SPRED2	3.06	CLU; MIR6843	−2.27
FA2H	3.05	OAS3	−2.27
DNAJB9	3.04	HELLS	−2.27
PGF	3.03	SFXN5	−2.27
TGFA	3.02	SLC29A1	−2.27
FOXN2	3.02	CD81	−2.27
EDEM1	3.01	NRP1	−2.27
EDNRA	3	AIM1	−2.28
NCF1	2.99	HIST1H3I	−2.28
ATF3	2.99	STARD13	−2.28
TMEM2	2.99	ACPP	−2.29
IL1RN	2.99	ITM2C	−2.29
SEC24D	2.99	ESCO2	−2.29
KDM6B	2.99	HACD2	−2.29
TGM5	2.98	PLK1	−2.29
ANKRD22	2.97	TLL1	−2.3

PRELID3B; ATP5E	2.97	PDPR	−2.3
CYB5R2	2.96	HMGN2	−2.3
DNAJB5	2.96	TM7SF3	−2.3
ACSL3	2.95	NMI	−2.3
STC2	2.94	CALD1	−2.3
MB21D1	2.94	SYT8	−2.3
DEFB103A; DEFB103B	2.94	F2RL2	−2.3
MREG	2.93	KIF13A	−2.3
TBC1D20	2.93	CKAP2	−2.31
TRIM8	2.92	RMI2	−2.31
GFPT1	2.92	KRT1	−2.31
IVL	2.91	AASS	−2.31
PHF1	2.91	SMO	−2.31
LPIN1	2.89	ID3	−2.31
SLC6A14	2.89	ABCC2	−2.31
CTH	2.88	BCAM	−2.32
ADAM8	2.87	YTHDC1	−2.32
ARPC1B	2.86	OLFML3	−2.32
EMP3	2.85	MAL2	−2.32
UBASH3B	2.85	CDC25C	−2.32
SLC16A3; MIR6787	2.85	CIT; MIR1178	−2.32
ZFAND5	2.84	SH3BGRL	−2.32
SLC2A6	2.84	MLLT3	−2.32
ABHD3	2.81	PCDH7	−2.33
FBXL12	2.81	ADAR	−2.33
HSPA13	2.8	GANC	−2.33
DHRS9	2.8	DLEU2; MIR15A; MIR16−1	−2.33
CDYL	2.79	PBK	−2.33
PLEK2	2.78	HIST2H2BF	−2.33
PLA2G4E	2.77	IL17RE	−2.33
SRPX2	2.76	CLSPN	−2.34
FAM83G	2.76	NLRP10	−2.34
EIF2B2	2.73	TMEM107; SNORD118	−2.34
RASA3	2.73	HEG1	−2.34
NIPAL1	2.72	SLC23A2	−2.34
DUSP16	2.72	KIF11	−2.34
SLC20A1	2.72	SLC39A2	−2.34
IER2	2.71	NALCN	−2.35
CPEB4	2.7	MANEA	−2.35
IRF2BPL	2.7	MMP13	−2.35
ANGPTL4	2.69	SLC5A1	−2.36
PLCXD1	2.69	AMZ2	−2.36
CHL1	2.67	FAM129A	−2.36
SOX9	2.67	TRIM59	−2.37
CHST11	2.67	CCNA2	−2.37
SC5D	2.67	IFI44	−2.37
ABCA12	2.66	PRR5L	−2.37
PSME4	2.66	ZNF845	−2.38

TRIM27	2.66	FBXW7	−2.38
PMEPA1	2.65	DEPDC4	−2.38
XPOT	2.65	BMPR1B	−2.39
AVPI1	2.65	BSCL2	−2.39
CLCF1	2.65	NCAPG2	−2.39
NUAK2	2.65	C8orf34	−2.39
DUSP16	2.64	MUC7	−2.4
FHDC1	2.64	BTG2	−2.4
NFKB2	2.64	NLRP10	−2.41
FAM210A	2.63	EPHA3	−2.41
DSCAM	2.63	LXN	−2.42
PGM3	2.63	MCM3	−2.42
PRNP	2.62	KIAA1644	−2.43
IL1R2	2.62	ALG8	−2.43
GJB5	2.62	NLRP2	−2.44
IFRD1	2.62	WNK1	−2.44
ARL4C	2.62	INPP5D	−2.44
CMTM7	2.61	GATA3	−2.44
CSF3	2.6	FOXM1	−2.44
FLG2	2.6	GTF2H5	−2.44
IARS	2.6	TYMS	−2.45
ODC1; SNORA80B	2.6	GJA1	−2.45
ME1	2.59	ISG15	−2.45
ASB1	2.59	MBNL2	−2.45
MAP3K9	2.59	MXRA5	−2.46
LRAT	2.58	EAF2	−2.46
RNF19B	2.58	TMEM123	−2.47
CCL20	2.58	TACC3	−2.47
SMOX	2.57	HACD3	−2.47
LIPG	2.57	PRICKLE1	−2.47
NIPA1	2.57	NCAPH	−2.48
NFKBIA	2.57	PSG6	−2.48
ALDH1A3	2.57	LPAR6	−2.48
SREBF1	2.57	VPS36	−2.48
TMEM2	2.56	RALGPS1	−2.48
SEMA4B	2.55	HIST1H2BF	−2.49
C10orf55	2.55	FIGN	−2.49
CYP1B1	2.55	CRYAB	−2.49
DAPP1	2.54	LAPTM4B	−2.49
SQSTM1	2.54	TMEM27	−2.49
MSANTD3−TMEFF1	2.53	ALG6	−2.49
WDR45; PRAF2	2.53	INPP5D	−2.49
WARS	2.52	GPSM2	−2.49
ACSL1	2.52	PIK3C2B	−2.5
TARS	2.52	CYP2J2	−2.5
ATP2B1	2.52	POLH	−2.52
SOCS2	2.52	TPRG1	−2.52
MPRIP	2.51	CDK1	−2.52

ACLY	2.51	HSD17B11	−2.52
PPARD	2.51	BLOC1S1	−2.52
EGR3	2.49	DDX58	−2.52
GLRX	2.49	CSPG5	−2.53
YOD1	2.49	PSG9	−2.53
SEMA3A	2.49	BNIPL	−2.53
NCAM1	2.49	TP53	−2.53
PHGDH	2.49	HIST1H2AL; HIST1H2BN	−2.53
SLFN5	2.49	EPHA4	−2.54
WSB1	2.48	BUB1	−2.54
MARS; MIR6758	2.48	BRCA1	−2.54
ARL5B	2.48	PNPO	−2.55
HPCAL1	2.48	HIST2H2AB	−2.55
DEDD2	2.48	NGFRAP1	−2.55
HERPUD1	2.46	SLC16A2	−2.55
ATG2A	2.46	HIST1H1C	−2.56
ZAK; pk	2.46	C1QL1	−2.56
GMPPB	2.45	SOX6; MIR6073	−2.57
SHROOM3	2.45	SLC2A12	−2.57
CARS	2.45	HIST2H2AA4; HIST2H2AA3	−2.57
HSD11B1	2.45	KRT13	−2.58
MIR22HG	2.45	DZIP3	−2.58
SLC37A2	2.45	TMEM14A	−2.58
TRPV3	2.44	SIDT2	−2.59
ABCA1	2.44	DEGS1	−2.59
SQSTM1	2.43	PIGS	−2.59
CA12	2.43	PSG4	−2.6
SKIL	2.42	CBR1	−2.6
FMNL2	2.42	UPK3B	−2.6
C9orf72	2.42	EPHX1	−2.6
RNF24	2.41	RBL1	−2.61
ALAS1	2.41	HIST1H4C	−2.61
ITGA5	2.4	FOXN3	−2.62
RANBP9	2.39	IFITM2	−2.62
SEMA6A	2.39	RRM2	−2.63
IDI1	2.39	B4GALT4	−2.63
ABL2	2.39	PLEKHF2	−2.63
KRT18	2.38	BRE	−2.63
JUND	2.38	RAD51	−2.63
PYGB	2.38	FZD1	−2.63
CSRNP1	2.38	IRAK1BP1	−2.64
LPXN	2.38	INPP5D	−2.64
RASA3	2.38	DNAL1	−2.64
CPNE8	2.38	DLL1	−2.64
CLDN12	2.37	HIST1H1D	−2.64
SAV1	2.36	HIST1H2BL	−2.65
ADGRE5	2.35	STARD5	−2.65
TDP2	2.35	KCND2	−2.66

CYP27B1	2.34	HIST1H4K	−2.66
PARD6B	2.34	HIST1H2BH	−2.66
DOCK4	2.34	TRIM69	−2.67
LHFPL2	2.34	MEIS1	−2.68
CDCP1	2.32	GRIA3	−2.68
PGD	2.32	NIPAL3	−2.68
SPDYA	2.32	HCFC1R1	−2.69
LOC643802	2.32	DLGAP5	−2.69
HMOX1	2.32	LFNG; MIR4648	−2.69
POLR3D	2.32	USP18	−2.71
SARS	2.32	HIST1H2AJ	−2.72
MEX3C	2.31	NREP	−2.72
CYTH1	2.31	FAM198B	−2.72
EID3	2.31	MYO3B	−2.73
LBH	2.31	CENPM	−2.73
LYAR	2.31	POGZ	−2.73
CTNS	2.3	CXADR	−2.74
CSGALNACT2	2.3	LGALSL	−2.74
GNE	2.29	PRSS12	−2.74
FZD8; MIR4683	2.29	VCL	−2.76
NCR3LG1	2.28	MSH2	−2.76
FEZ1	2.28	HIST1H2AM; HIST1H3J	−2.76
AARS	2.28	MMP2	−2.77
ZNF805	2.28	TRANK1	−2.78
CEBPB	2.28	BDH2	−2.78
PMM2	2.27	NNT	−2.78
YARS	2.27	WDR66	−2.78
SPINK1	2.27	E2F8	−2.79
OSBP2	2.27	HMGB2	−2.79
SLC4A7	2.26	CBX5; MIR3198−2	−2.79
SLC2A6	2.26	CSRP2	−2.8
ELK3	2.26	TMEM255A	−2.8
PNPLA8	2.26	CXCL16	−2.81
ENTPD3	2.26	LAMP3	−2.81
MXD1	2.26	UGT1A1	−2.81
SREK1IP1	2.26	PLSCR1	−2.81
MST1R	2.26	HIST1H3B	−2.82
ITGB4	2.26	KIAA1107	−2.83
MAFF	2.25	BLNK	−2.83
CEBPA	2.25	KIAA0101; CSNK1G1	−2.83
SQRDL	2.24	SLC47A2	−2.83
HAUS6	2.24	SLC16A9	−2.84
FBXO33	2.24	LCP1	−2.84
TNFRSF25	2.24	CERS6	−2.84
HR	2.23	MICAL3	−2.86
DUSP5	2.23	S1PR5	−2.86
DUSP16	2.22	ARL15	−2.86
PPP1R15A	2.22	EPGN	−2.87

SEC24A	2.22	FADS3	−2.87
TUBGCP3	2.21	WNT10A	−2.88
KBTBD2	2.21	EIF2AK2	−2.88
TRIOBP; NOL12	2.21	SHTN1	−2.89
TRIML2	2.21	TXNIP	−2.91
SETBP1	2.21	CCL28	−2.91
HMGCS1	2.21	CCNE2	−2.91
IER5	2.21	DDX60L	−2.92
NAMPT	2.21	FGFR3	−2.93
CYTH2	2.2	SSBP2	−2.93
PLEKHM1; MIR4315−1	2.2	KDM2B	−2.93
ZNF165	2.2	SLC6A11	−2.95
TOP1	2.2	ZSCAN31	−2.95
DEFB103A	2.2	SLC6A2	−2.96
TMEM189	2.19	EBPL	−2.96
MED8	2.19	MLLT3	−2.97
UGDH	2.19	HIST1H2AG	−2.99
ARFIP2	2.19	PCNA	−3
ZFAND1	2.19	VAV3	−3
ZBTB10	2.19	MX2	−3.01
JOSD1	2.19	FAM26E	−3.01
LYN	2.19	CCBE1	−3.01
TMED5	2.19	SKP2	−3.01
GPX2	2.18	IFT74	−3.02
RGS12	2.18	TMEM138	−3.05
ATP2C2	2.18	ARV1	−3.06
RAB31	2.18	CYP39A1	−3.06
KLF7	2.18	PROS1	−3.06
FGF2	2.17	LGR4	−3.06
ULK1	2.17	B4GALNT2	−3.07
NRBF2	2.17	DTL	−3.07
WNT5A	2.17	OR5P2	−3.07
IFRD2	2.17	SCD5	−3.09
PTPN12	2.17	SLC26A2	−3.09
RNF144B	2.16	CD86	−3.12
GTPBP2	2.16	MAFTRR	−3.13
SNRK	2.16	SLC9A9	−3.14
EIF2AK3	2.16	HIST1H3F	−3.15
NTN4	2.16	SLC29A3	−3.19
SCML1	2.15	LYPD6B	−3.19
DUSP16	2.15	ETS2	−3.22
SH2D3A	2.15	TLR5	−3.23
PRKAG2	2.15	CRABP2	−3.23
TIMM22	2.15	KRT77	−3.23
ZNF432	2.14	STIL	−3.26
COQ10B	2.14	CAV1	−3.26
NFAT5	2.14	NR2F2	−3.27
ILF2	2.14	ENDOD1	−3.29

VPS37B	2.13
C15orf57; MRPL42P5	2.13
SLC30A1	2.13
G0S2	2.13
ARHGEF18	2.13
TOM1L1	2.13
MIR17HG; MIR17	2.12
BAK1	2.12
RNASE7	2.12
RELL2	2.12
DHCR7	2.12
CRLF3	2.12
SMIM15	2.12
SHB	2.11
GJB3	2.11
SH3PXD2B	2.11
RPS6KA3	2.11
QKI	2.11
ENTPD6	2.11
YDJC	2.11
HK2	2.1
SPIRE1	2.1
NDEL1	2.1
STK17A	2.1
ARCN1	2.1
NANS	2.1
MAP2K1	2.1
RSL24D1	2.1
ST3GAL1	2.1
IL36G	2.09
SH3KBP1	2.09
CDC42EP2	2.09
LPAR3	2.09
SLC52A2	2.09
SMG9	2.09
VIM	2.09
PIGA	2.09
TIPARP	2.09
NFIL3	2.09
ZFAND2A	2.09
UGCG	2.09
UBE2R2	2.08
TNIP1	2.08
PELI1	2.08
HSD17B12	2.08
RAPH1	2.08
LTBR	2.08
MARVELD2	2.08
MCM5	−3.31
IFIT3	−3.31
ERG	−3.31
MOB3B	−3.33
RBMS3	−3.33
HIST1H2BI	−3.34
IFIH1	−3.35
MCM6	−3.36
VWA5A	−3.4
STEAP4	−3.41
IFITM3	−3.44
STMN1; MIR3917	−3.45
SESN3	−3.49
GSTM4	−3.49
MFAP5	−3.5
SPOCD1	−3.5
FGFR2	−3.51
TRAM2	−3.52
ZNF608	−3.52
HIST1H4J	−3.53
HACD4	−3.55
RNF141	−3.57
RAB7B	−3.57
NID1	−3.58
TOP2A	−3.6
HIST1H3H	−3.6
CACNG4	−3.61
HIST2H2AA3; HIST2H2AA4	−3.61
GPR1	−3.62
SPOCK1	−3.62
NUSAP1	−3.63
CYBRD1	−3.67
DTX3L	−3.67
DKK3	−3.69
EFEMP1	−3.69
SYNPO	−3.71
FAM171B	−3.74
CYP4B1	−3.74
IFFO2	−3.77
HIST1H1B	−3.77
PAQR7	−3.77
ERP27	−3.77
EXO1	−3.78
BCL11A	−3.8
PHACTR2	−3.82
VIT	−3.82
HIST1H2BM	−3.84
ENC1	−3.85

ARF4	2.08	ANLN	−3.89
CXXC1	2.08	SAMHD1	−3.9
ATG101	2.08	PARP14	−3.91
EIF2A	2.08	ENAH	−3.92
HMGA2	2.08	HIST1H2AI	−3.93
CPT1A	2.07	KANK4	−3.95
TMEM135	2.07	SP100	−3.96
DCBLD2	2.07	MAF	−3.99
PPP1R18	2.07	MATN2	−3.99
RICTOR	2.07	FAM111B	−4.01
PGM1	2.06	HIST1H2AB	−4.04
PTPN9	2.06	ADAMTS1	−4.05
LONRF1	2.06	DAPK1	−4.06
PPP1R10	2.06	ABI3BP	−4.08
MFSD2A	2.06	C1S	−4.1
SLC33A1	2.06	ACSF2	−4.11
GRB10	2.06	WNT4	−4.12
ITGB5	2.05	CPA4	−4.12
ARHGEF4	2.05	KITLG	−4.13
ABHD17C	2.05	C21orf91; C21orf91−OT1	−4.13
VASP	2.05	ELAVL2	−4.14
LSS	2.05	IFI44L	−4.17
TIPARP	2.04	CTSV	−4.22
DOC2A	2.04	HIST1H3G	−4.24
EHD4	2.04	EPSTI1	−4.25
ACAT2	2.04	GBP6	−4.26
SAMD8	2.04	HIST1H3A	−4.32
MPHOSPH6	2.04	FAM134B	−4.32
LAMB3; MIR4260	2.04	S1PR1	−4.33
SEC23A	2.03	C1orf21	−4.33
USP2	2.03	GBP2	−4.38
DYRK3	2.03	UBE2L6	−4.4
MAP4K4	2.03	KMO	−4.59
SLC39A7	2.03	COBLL1	−4.61
B3GNT3	2.03	CROT	−4.65
GAREM1	2.03	MTUS1	−4.72
AVL9	2.03	PPP2R2B	−4.72
MET	2.03	FRK	−4.73
PXN	2.03	C1R	−4.76
PTAFR	2.02	SP110	−4.76
RTTN	2.02	CA2	−4.78
MYD88	2.02	ADM	−4.92
LIF	2.02	GLIPR1	−4.95
CAB39L	2.02	NPNT	−4.99
MBOAT2	2.01	CYB561A3	−4.99
SPRED1	2.01	SAMD9L	−5.13
C1QTNF1	2.01	NMU	−5.16
STK40	2.01	CYR61	−5.21

MID1IP1	2.01
PREB	2.01
SRP19; ZRSR1	2
EMP1	2
GMEB1	2
MESDC1	2

EPPK1	−5.27
HIST2H3A; HIST2H3C	−5.29
FAT4	−5.3
CTGF	−5.36
MAP2	−5.48
TRIM22	−5.51
HIST2H3A	−5.6
LOX	−5.72
OTUD1	−5.72
SEMA5A	−5.74
WFDC12	−5.76
IFNK	−5.91
EDN1	−6.07
SAMD9	−6.08
BBOX1	−6.38
PARP9	−6.51
SCARA3	−6.58
TGFB2; TGFB2−OT1	−6.6
TPM1	−6.65
IFI27	−6.8
TNFSF10	−6.92
OAS2	−6.97
IFITM1	−7.15
MYL9	−7.24
FBXO32	−7.55
CCDC80; LINC01279	−7.88
STAT1	−8.01
TLR3	−8.02
OAS1	−8.27
DDX60	−8.53
FILIP1L	−8.62
PLK2	−8.97
OLFML2A	−9.52
PSG5	−10.16
TAGLN	−10.71
GBP1	−11.06
CXCL14	−15.76
IGFBP3	−19.59
IFI6	−20.11
IFIT1	−26.22
MX1	−36.58

Supplementary Table S2

Antibody array data:

**upregulated during AC EF stimulation**		**downregulated during AC EF stimulation**	
Protein	***log FC***	***Protein***	***log FC***
TP53	0.32	ENTPD1	−0.26
PDCD1LG2	0.35	IL10	−0.25
APP	0.31	TNFRSF9	−0.28
CASP3	0.31	S100P	−0.39
CFLAR	0.52	HMGA1	−0.42
AKT3	0.52	VCAM1	−0.31
ETS1	0.30	CASP3	−0.20
NT5E	0.43	ACTN1	−0.23
ATM	0.78	S100B	−0.33
NBN	0.31	BECN1	−0.38
IDO1	0.33	CDC25C	−0.21
JAK3	1.23	APBA1	−0.23
SOX9	0.46	GP1BA	−1.03
VTN	0.00	CD47	−0.26
CXCL13	0.31	MPO	−0.40
FOS	0.62	CD38	−0.26
CD14	0.66	SELE	−0.24
CD28	0.45	IL37	−0.21
CD44	0.44	IFNG	−0.40
PTPRC	0.96	IL10	−0.67
CD53	0.36	CCL5	−0.46
ENG	0.35	CD86	−0.22
PRTN3	0.30	HGF	−0.33
CD44	0.33	CSF2	−0.23
CD44	0.39	IL7R	−0.22
CD47	0.85	AKT3	−0.25
B3GAT1	0.30	RNF141	−0.24
IGLC1	1.13	TMEM54	−0.20
IL2RA	0.61	UNC93B1	−0.20
MAPK1	0.67	UXS1	−0.24
ISOC2	0.35	TNFSF10	−0.25
NAA50	0.32	MAPK9	−0.36
RP9	0.91	AQP1	−0.26
DYNC1I2	0.60	PIGT	−0.27
ERBB2	0.31	FGF2	−0.20
CHL1	0.39	IL10	−0.26
RANBP2	0.36	IL15	−0.22
FABP5	0.73	HSPB1	−0.24
PIGK	0.40	ANXA1	−0.24
PIGK	0.35	GSTM1	−0.21
GLO1	0.46	RAC1	−0.23
GLO1	0.45	MBD1	−0.24

GSTM1	0.66
ODC1	0.33
ODC1	0.49
S100A4	0.34
MDH1	0.30
DDIT3	0.37
HSPD1	0.61
FAF1	0.47
IDO1	0.85
CASP9	0.30
MMP7	0.39
MUC2	0.81
SORL1	0.94
FLOT1	0.47
MMP1	0.41
RAD51C	0.90
CUL2	0.68
RHBDF1	0.35
ECHS1	0.40
COL15A1	0.32
ENO1	0.96
MMP12	0.42
MAPK4	0.37
CCR7	0.85
ERBB2	0.59
GRB10	0.47
CHL1	0.38
CCKBR	0.52
CCR7	0.29
CFLAR	0.50
IRF4	0.27
SERPINA9	0.25
CCND2	0.23
STAT3	0.24
SH3BP5	0.20
PAX5	0.21
CHEK2	0.25
VTCN1	0.29
FABP2	0.23
THBS3	0.21
KLK3	0.26
ICAM1	0.23
FAS	0.40
CD99	0.21
ENG	0.20
CCL11	0.21
IFNG	0.21
IL7	0.21

FABP2	−0.42
S100A2	−0.24
PDLIM1	−0.23
HSPD1	−0.86
MUC17	−0.24
TACSTD2	−0.39
OTUB1	−0.37
AOX1	−0.44
AGR2	−0.26
S100A6	−0.53
ICAM1	−0.49
EIF2B1	−0.23
HSP90B1	−0.24
TIMP2	−0.28
KDR	−0.23
AMFR	−0.60
SPINT2	−0.37
TNF	−0.38
SORL1	−0.27
THOC1	−0.25
PDCD2	−0.54
CNN2	−0.41

NTF4	0.24
ITGA5	0.29
SELP	0.29
CCL8	0.02
MAP2K4	0.30
UBE2T	0.23
PGAM5	0.20
MAPK12	0.23
TNFSF14	0.21
CDC34	0.27
NME2	0.27
BCL2L2	0.22
ANXA1	0.25
ODC1	0.29
PPP2R5D	0.24
GSTM1	0.25
S100A2	0.25
PSMD4	0.27
TOP1	0.27
OGG1	0.24
NME1	0.21
CHKA	0.22
TDP2	0.25
NCOR1	0.26
CD302	0.22
BNIP3	0.26
CDKN1C	0.24
IGFBP7	0.28
DKK3	0.24
ODC1	0.25
TAF12	0.24
ARHGAP6	0.26
HSPA1A	0.21
MET	0.20
MMP10	0.29
SF3B4	0.26
CAV3	0.28
PI3	0.29
PTPRCAP	0.20
HMMR	0.22
PIR	0.20
MIF	0.26
GCHFR	0.33
MUC5B	0.20
SLC16A6	0.24
CCT7	0.25
RPL10A	0.36
